# *BRCA1* mutation promotes sprouting angiogenesis in inflammatory cancer-associated fibroblast of triple-negative breast cancer

**DOI:** 10.1038/s41420-023-01768-5

**Published:** 2024-01-05

**Authors:** Chae Min Lee, Yeseong Hwang, Jae Woong Jeong, Minki Kim, Janghee Lee, Soong June Bae, Sung Gwe Ahn, Sungsoon Fang

**Affiliations:** 1 Graduate School of Medical Science, Brain Korea 21 Project, Yonsei University College of Medicine, Seoul, 03722 Republic of Korea; 2 Department of Biomedical Sciences, Gangnam Severance Hospital, Yonsei University College of Medicine, Seoul, 03722 Republic of Korea; 3 Department of Medicine, Yonsei University College of Medicine, Seoul, 03722 Republic of Korea; 4 Department of Surgery, Sacred Heart Hospital, Hallym University, Dongtan, 18450 Republic of Korea; 5 Department of Medicine, Yonsei University Graduate School, Seoul, 03722 Republic of Korea; 6 Department of Surgery, Gangnam Severance Hospital, Yonsei University College of Medicine, Seoul, 06273 Republic of Korea; 7 Institute for Breast Cancer Precision Medicine, Yonsei University College of Medicine, Seoul, 06273 Republic of Korea; 8 Chronic Intractable Disease for Systems Medicine Research Center, Yonsei University College of Medicine, Seoul, 03722 Republic of Korea; 9 Severance Institute for Vascular and Metabolic Research, Yonsei University College of Medicine, Seoul, 03722 Republic of Korea

**Keywords:** Breast cancer, Breast cancer

## Abstract

Triple-negative breast cancer (TNBC) is an aggressive breast cancer subtype with inferior outcomes owing to its low treatment response and high invasiveness. Based on abundant cancer-associated fibroblasts (CAFs) and frequent mutation of breast cancer-associated 1 *(BRCA1)* in TNBC, the characteristics of CAFs in TNBC patients with *BRCA1* mutation compared to wild-type were investigated using single-cell analysis. Intriguingly, we observed that characteristics of inflammatory CAFs (iCAFs) were enriched in patients with *BRCA1* mutation compared to the wild-type. iCAFs in patients with *BRCA1* mutation exhibited outgoing signals to endothelial cells (ECs) clusters, including chemokine (C-X-C motif) ligand (CXCL) and vascular endothelial growth factor (VEGF). During CXCL signaling, the atypical chemokine receptor 1 (ACKR1) mainly interacts with CXCL family members in tumor endothelial cells (TECs). *ACKR1*-high TECs also showed high expression levels of angiogenesis-related genes, such as *ANGPT2*, *MMP1*, and *SELE*, which might lead to EC migration. Furthermore, iCAFs showed VEGF signals for *FLT1* and *KDR* in TECs, which showed high co-expression with tip cell marker genes, including *ZEB1* and *MAFF*, involved in sprouting angiogenesis. Moreover, *BRCA1* mutation patients with relatively abundant iCAFs and tip cell gene expression exhibited a limited response to neoadjuvant chemotherapy, including cisplatin and bevacizumab. Importantly, our study observed the intricate link between iCAFs-mediated angiogenesis and chemoresistance in TNBC with *BRCA1* mutation.

## Introduction

Triple-negative breast cancer (TNBC) is characterized by a lack of estrogen receptor, progesterone receptor, and human epidermal growth factor 2 receptor protein and comprises 15–20% of breast cancer cases [1, 2]. TNBC is the most aggressive type of breast cancer owing to its low response to treatment and high invasiveness [3]. Approximately 46% of TNBC patients present distant metastasis in the third year after diagnosis [4–6]. TNBC usually spreads to diverse metastatic sites, including the brain and lungs, and is more likely to exhibit visceral metastasis than other breast cancer subtypes within 5 years of diagnosis [7]. Therefore, investigating the molecular mechanisms to suppress TNBC metastasis is essential.

Breast cancer-associated (BRCA) tumor suppressor genes play crucial roles in DNA damage repair, recombination, cell-cycle arrest, apoptosis, and transcription regulation [8]. Mutations in *BRCA* genes are associated with a high risk of developing breast cancer [9, 10]. Approximately 55–72% of women carrying a harmful *BRCA1* gene variant and around 45–69% of those with a harmful *BRCA2* gene variant are expected to develop breast cancer during their 70-80 years of life [11, 12]. Metastasis of breast cancer occurs more frequently in carriers of *BRCA1* mutation, often manifesting as lung metastases and distant lymph node involvement [13]. In fact, over 75% of female breast cancer patients with *BRCA1* mutation exhibit a TNBC phenotype [14]. Patients with *BRCA1* mutation are more prone to develop TNBC compared to patients with *BRCA2* mutation and wild-type, with a prevalence of 5–10% [15, 16].

Recently, single-cell RNA-sequencing (scRNA-seq) has emerged as a frequently employed technique to investigate the cellular landscape and uncover novel aspects of various cancers, including breast cancer and prostate cancer [17, 18]. The studies comprehensively characterized the transcriptional atlas or significant gene expression of breast cancer. Furthermore, numerous studies have explored distinct tumor factors and cellular diversities in breast cancer stemming from various oncogenic events [19–22]. One study conducted scRNA-seq analysis to compare wild-type and mutant cells across diverse cancer types [23].

The scRNA-seq technique has recently revealed the complexity and heterogeneity of the tumor microenvironment (TME) in diverse cancers. Many components, including cancer-associated fibroblasts (CAFs), endothelial cells (ECs), and immune cells, exist in TME. CAFs are the most abundant cells and play key roles in the TME, mainly stimulating tumor growth, tumor progression, and drug resistance [24]. Several types of CAFs can be segregated based on their specific cell markers, such as inflammatory-CAFs (iCAFs) and myofibroblastic-CAFs (myCAFs) [25]. It was reported that breast cancer patients with *BRCA1* mutation have a high-level iCAF signature [26]. iCAFs have diverse inflammatory signaling pathways such as interferon γ response, tumor necrosis factor/nuclear factor kappa-β, interleukin (IL)-2/signal transducer and activator of transcription (STAT) 5, and IL-6/Janus kinase (JAK)/STAT3. The IL-1/LIF/JAK/STAT pathway can activate iCAFs, leading to ECM deposition and immune suppression promoting cancer initiation and progression. iCAFs secrete many members of the CXCL and IL family [27, 28], leading to epithelial-mesenchymal transition and angiogenesis. However, the pathogenic role of the *BRCA1* mutation/iCAFs axis in TNBC has rarely been investigated.

This study revealed the role of iCAFs in tumorigenesis in TNBC patients with *BRCA1* mutation by scRNA-seq analysis. We confirmed that iCAFs preferentially reside in the fibroblast cluster and display outgoing signaling towards EC clusters, such as *CXCL* and *VEGF*, which might promote EC migration and angiogenesis in TNBC patients with *BRCA1* mutation. Finally, the data implied that TNBC patients with *BRCA1* mutation who had high levels of iCAFs and tip cells involved in angiogenesis exhibited resistance to neoadjuvant chemotherapy, including cisplatin and bevacizumab. Our data suggest that TNBC patients with *BRCA1* mutation and high levels of the iCAFs-related genes can be insensitive to anti-angiogenic therapy.

## Results

### TNBC patients represent a robust expression of CAF signature

**Fig. 1:**
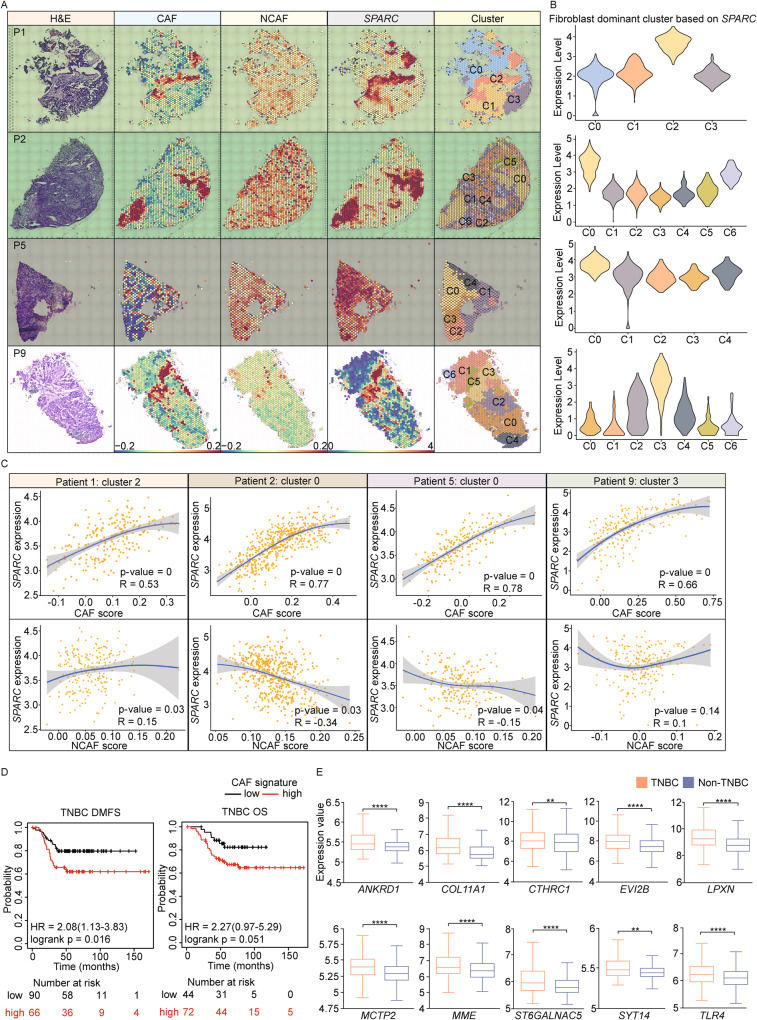
CAFs prominently reside in TNBC patients. **A** Visium spatial gene expression data shows that the expression of CAF and NCAF signature in the fibroblast dominant area based on the marker gene *SPARC*. The GEO dataset was acquired from GSE210616. **B** Violin plots show the fibroblast dominant clusters based on *SPARC* expression in each patient. The highest clusters (C2 in P1, C0 in P2, C0 in P5, and C3 in P9) are highlighted in yellow. **C** Scatter plots illustrate the correlation between CAF or NCAF signature and *SPARC* in the fibroblast dominant cluster identified in (**B**). Four patients who represented the highest difference between CAF and NCAF signatures were named 1, 2, 5, and 9 (P1, P2, P5, P9) among 22 TNBC patients. R-values were calculated using the ‘FeatureScatter’ function in R, while *p*-values were calculated using the Social Science Statistics website (https://www.socscistatistics.com/pvalues/pearsondistribution.aspx). **D** The Kaplan–Meier curves show that TNBC patients with higher expression levels of CAF genes show poor prognosis in the DMFS (left) and OS (right). HR values and log-rank *p-*values were calculated using the KM Plotter database. **E** Box plots demonstrate that TNBC patients (*n* = 1980) have higher expression levels of CAF genes compared to other types of non-TNBC groups (*n* = 144). *p*-values were computed on the BCIP website (**p < 0.01, ****p < 0.0001).

The correlation between TNBC and the CAF signature was investigated using spatial gene expression data from the Visium platform obtained from the GSE210616 dataset. Among the 22 patients with TNBC, we observed the expression of the CAF and non-CAF (NCAF) signature in the fibroblast area based on the fibroblast marker gene *SPARC*. To identify the CAF abundance in TNBC in more detail, we discriminated the fibroblast dominant cluster based on *SPARC* and observed the correlation between *SPARC* and CAF or NCAF signature in each patient [29]. Although the NCAF signature in 4 patients exhibited a positive correlation with *SPARC*, the CAF signature in 14 patients showed a significant positive correlation with *SPARC* (Figs. 1A–C and S1A–C). Furthermore, the high CAF signature group in TNBC patients showed poor prognosis in terms of distant metastasis-free survival (DMFS) and overall survival (OS).

However, other subtypes of breast cancer showed no significant differences in DMFS, indicating that higher CAF signature specifically affects the survival of TNBC patients (Figs. 1D and S2). We also confirmed TNBC patients exhibited significantly higher expression levels of CAF-related genes, such as *ANKRD1*, *COL11A1*, *CTHRC1*, *EVI2B*, *LPXN*, *MCTP2*, *MME*, *ST6GALLNAC5*, *SYT14*, and *TLR* compared to non-TNBC patients (Fig. 1E). In summary, our data suggest that patients with TNBC display a highly upregulated CAF signature, which may be a risk factor for poor prognosis.

### Single-cell analysis revealed that iCAF signaling is dominant in TNBC patients with *BRCA1* MT

**Fig. 2:**
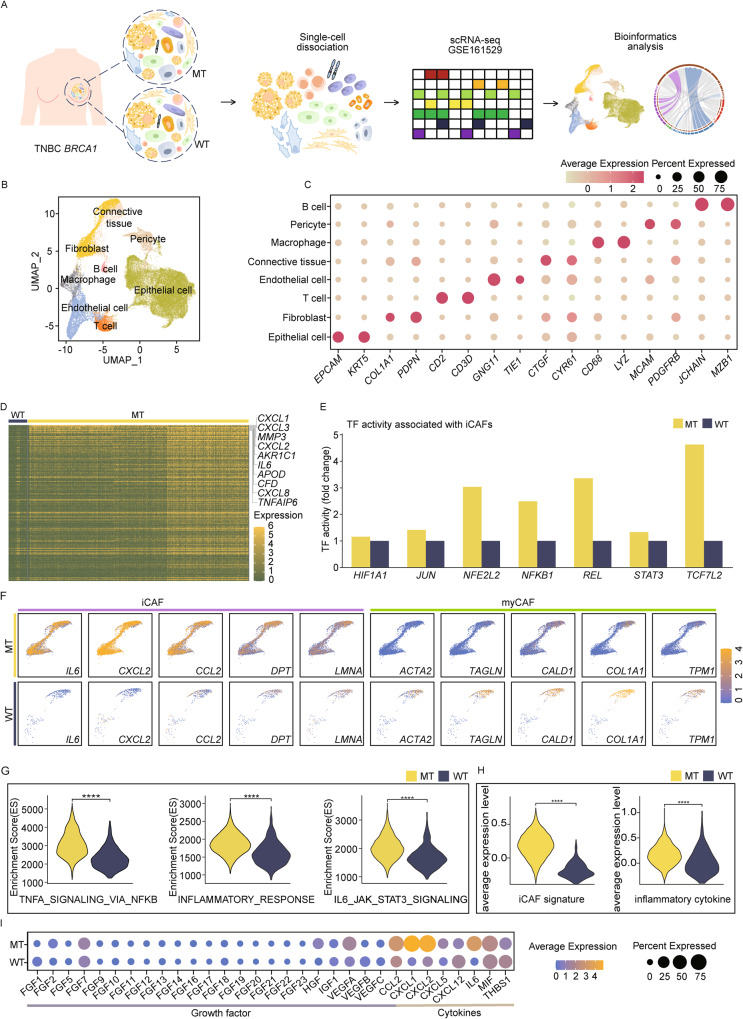
TNBC *BRCA1* MT significantly displays iCAFs phenotype. **A** Experimental design shows scRNA-seq analysis of integrated TNBC *BRCA1* MT (n = 4) and WT (n = 4). The dataset was obtained from GSE161529. **B** UMAP plot exhibits eight integrated clusters. **C** Dot plot represents marker genes for annotating eight clusters in B. The scale and dot size mean the average gene expression and percent expression, respectively. **D** The heatmap shows the top 10 upregulated DEGs in TNBC *BRCA1* MT fibroblast were associated with iCAFs. **E** Bar graphs represent the activity of iCAF-related transcription factors, which was higher in TNBC *BRCA1* MT compared to WT. **F** Feature plots show that iCAFs and myCAFs-related genes are highly expressed in TNBC *BRCA1* MT and WT fibroblast clusters, respectively. **G** Violin plots illustrate that diverse pathways associated with iCAFs, such as tumor necrosis factor-α signaling via nuclear factor kappa-β, inflammatory response, and IL-6/JAK/STAT3 signaling, are enriched in TNBC *BRCA1* MT fibroblasts compared to WT fibroblasts. **H** Violin plots show that the expression level of iCAFs and inflammatory cytokine signature was higher in TNBC *BRCA1* MT than in WT. The ‘p.adjust’ function in R was utilized to derive the *q*-values. **I** Dot plot displays the expression level of several growth factors and cytokines in TNBC *BRCA1* MT and WT. The expression level of *IL-6* and *CXCL* was significantly higher in the TNBC *BRCA1* MT. *q*-values in **G**, **H** were obtained using the ‘p.adjust’ function in R (****q < 0.0001).

Because *BRCA1* mutation occurs frequently in TNBC and diverse CAF phenotypes exist in cancer, we next performed scRNA-seq analysis to compare the characteristics of CAF between patients with TNBC *BRCA1* mutation type (MT) and wild type (WT) (Fig. 2A). During analysis of total 35,234 cells, we identified a diverse range of cell types including epithelial cells (*EPCAM*, *KRT5*), fibroblast (*COL1A1*, *PDPN*), T cell (*CD2*, *CD3D*), endothelial cell (*GNG11*, *TIE1*), connective tissue (*CTGF, CYR61*), macrophage (*CD68*, *LYZ*), pericyte (*MCAM*, *PDGFRB*), and B cell (*JCHAIN*, *MZB1*) (Fig. 2B, C and S3A, B) [30–36]. We also confirmed that the cell type proportion was heterogeneous in each patient, and among them, the fibroblast proportion was higher in MT (Fig. S3C). To further compare the CAF composition between MT and WT, we subclustered the fibroblasts (Fig. 2D). During the analysis of differentially expressed genes (DEGs) between MT and WT, we identified that the top 10 DEGs, including *CXCL1, CXCL3*, *MMP3*, *CXCL2*, *AKR1C1*, *IL-6*, *APOD*, *CFD*, *CXCL8*, and *TNFAIP6*, are associated with inflammation-related genes (Fig. 2D). Additionally, the expression levels of iCAFs genes, such as *IL-6, CCL2* were highly upregulated in MT. In comparison, the expression levels of myCAFs genes were prominently dominant in WT (Fig. 2E). When we compared transcription factor (TF) activity between MT and WT fibroblast, MT exhibited higher TF activity associated with iCAFs, including *HIF1A1, JUN, NFE2L2, NFKB1, REL*, and *STAT3* (Fig. 2F). Single-cell gene set enrichment analysis also showed iCAFs signaling, such as IL-6/JAK/STAT3, tumor necrosis factor-α signaling via nuclear factor kappa-β, and the inflammatory response was enriched in the MT group (Fig. 2G). Furthermore, the iCAF signature and average expression levels of inflammatory cytokines were notably higher in MT (Fig. 2H), suggesting that iCAFs preferentially reside in *BRCA1* MT TNBC. iCAFs secrete diverse growth factors and cytokines into other TME or tumors [37, 38]. Therefore, we compared the expression levels of growth factors and cytokines between MT and WT. MT exhibited significantly higher expression levels of several growth factors and cytokines, including *VEGFA*, *CCL2*, *IL-6*, and the CXCL family, than WT (Fig. 2I). Altogether, our data imply *BRCA1* MT and WT TNBC patients exhibit distinct CAF compositions and especially iCAFs features are more prominent than myCAFs in MT.

### iCAFs mainly exhibit outgoing signals to endothelial cells in TNBC *BRCA1* mutation

**Fig. 3:**
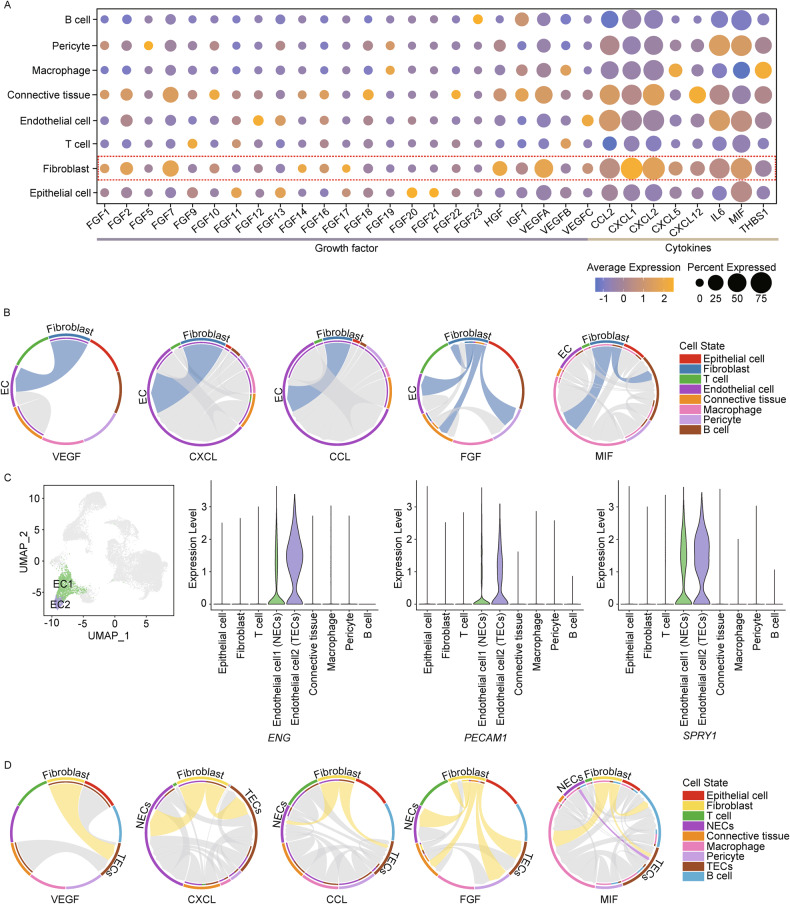
ECs are major incoming target cells from iCAFs in TNBC *BRCA1* MT. **A** Dot plot indicates the average and percent expression of growth factor and cytokines across the total 8 clusters in the TNBC *BRCA1* MT. **B** Chord diagram illustrates that fibroblasts mainly exhibit outgoing signaling toward ECs, including five signals: VEGF, CXCL, CCL, FGF, and MIF. **C** UMAP and violin plots show that the EC2 cluster had high expression of TEC-related genes such as *ENG*, *PECAM1*, and *SPRY1* compared to the EC1 cluster. **D** Chord diagrams show predicted interaction pathways between fibroblast clusters and two types of ECs mentioned in (**B**).

Next, we investigated the crosstalk between iCAFs and other cell types in all clusters. We first identified several significantly upregulated expression levels of *FGF7*, *VEGFA*, and *CXCL1*, *CXCL2* in fibroblast cluster (Fig. 3A). Interestingly, these chemical messengers in fibroblasts mainly influence the ECs (Fig. 3B). Therefore, we further segregated ECs into tumor ECs (TECs) and normal ECs (NECs) using the TEC marker genes *ENG*, *INSR*, *PECAM1*, and *SPRY1* (Fig. 3C). When we next again observed the signaling pathways mentioned in Fig. 3B, all the chord diagrams showed outgoing signals to TECs or NECs despite different patterns according to signaling pathways. Notably, we found that VEGF and CXCL signaling from fibroblast achieved powerful communication with TECs compared to other types of signaling pathways (Fig. 3D). Collectively, our data indicated that ECs are the major components of iCAFs-induced signaling and may exert tumorigenic effects in *BRCA1* MT TNBC.

### TECs are a major target of incoming signaling from iCAFs in TNBC *BRCA1* MT

**Fig. 4:**
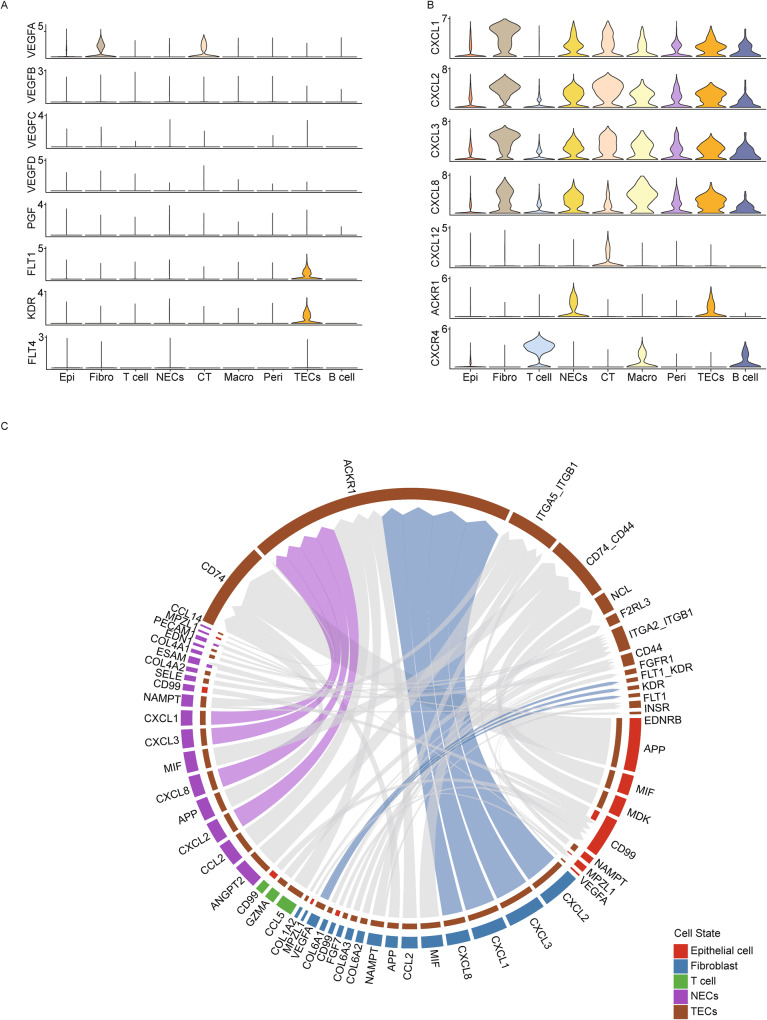
TECs participate in CXCL and VEGF signaling with iCAFs in TNBC *BRCA1* MT. **A** Violin plots indicate *VEGFA* is highly expressed in fibroblasts in the VEGF family, whereas the receptor *FLT1* (*VEGFR1*) and *KDR* (*VEGFR2*) are only expressed in TECs. **B** Violin plots show diverse CXCL families, including *CXCL 1*, *2*, *3*, and *8*, which are expressed in all clusters, including fibroblast. In contrast, the receptor *ACKR1* is only expressed in two types of ECs. **C** Chord diagram displays that TECs (brown) present prolific interactions with fibroblast (blue) and NECs (purple) in terms of the CXCL family and *VEGFA*. Each gene targets the *ACKR1*, *FLT1*, and *KDR* in TECs.

Based on the strong expression levels of the VEGF and CXCL family and outcoming patterns from fibroblast to ECs in MT (Figs. 2I and 3A, D), we next specifically analyzed VEGF and CXCL signaling between iCAFs and ECs. Remarkably, the fibroblast cluster exhibited strong expression levels of *VEGFA* but not *VEGFB*, *C* and *VEGFR*, *FLT1* and *KDR* were expressed only in TECs (Fig. 4A). In addition, fibroblasts showed the highest expression of *CXCL1*, *CXCL*2, *CXCL3*, *CXCL8*, and their receptor *ACKR1* was expressed in both NECs and TECs (Fig. 4B). This finding led us to investigate whether CXCL family and *VEGFA* interact with *ACKR1*, *FLT1*, or *KDR* using a chord diagram. Interestingly, we found that signals of CXCL family with *ACKR1* were not detected in NECs, and receptor-ligand interaction robustly occurred between iCAFs and TECs, including CXCL family and VEGF signaling (Fig. 4C). In summary, our data imply that TECs play an important role in tumorigenic effects by communicating with iCAFs in *BRCA1* MT TNBC.

### *ACKR1* high TECs provoke migration in response to CXCL signaling

**Fig. 5:**
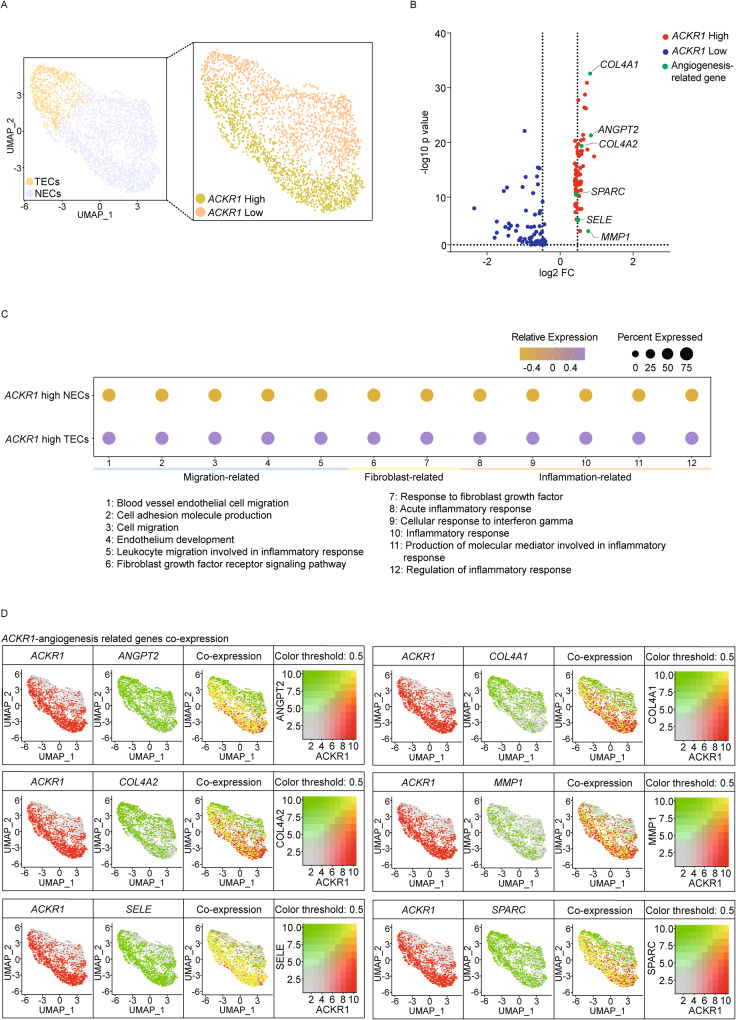
*ACKR1* high TECs induce angiogenesis via communicating with the CXCL family in *BRCA1* MT TNBC. **A** Feature plot shows ECs were categorized into TECs and NECs (left) and again segregated into four clusters based on *ACKR1* expression level (right) in TNBC *BRCA1* MT. **B** Volcano plot illustrates several angiogenic process-related genes were enriched in *ACKR1*-high TECs (|log_2_ FC| ≥ 0.4, *p* < 0.05). **C** Dot plot presents *ACKR1*-high TECs that exhibit higher expression levels related to migration, fibroblast, and inflammation compared to *ACKR1*-high NECs. **D** Combined feature plots represent high co-expression of *ACKR1* and angiogenesis-related genes in the TECs cluster.

To specifically investigate the CXCL family/*ACKR1* axis in ECs, we subdivided TECs and NECs into four distinct groups based on *ACKR1* and TECs marker genes: *ACKR1* high TECs, *ACKR1* low TECs, *ACKR1* high NECs, and *ACKR1* low NECs (Fig. 5A). In upregulated DEGs in *ACKR1* high TECs compared to *ACKR1* high NECs, we identified diverse angiogenic process-related genes including loosening of blood vessel *ANGPT2*, immune adhesion *SELE*, ECM remodeling *COL4A1*, *COL4A2*, *MMP1*, and *SPARC*. (Fig. 5B) When we compared *ACKR1* high TECs and *ACKR1* high NECs in the GO pathway enrichment analysis, pathways associated with migration, fibroblast receptor, and inflammation were all enriched in *ACKR1* high TECs (Fig. 5C), indicating the possible involvement of *ACKR1* high TECs in migration. Moreover, co-expression of *ACKR1* and TEC marker genes was evident in both *ACKR1* high TECs clusters (Fig. 5D). Our data suggest that ECs migration via the CXCL family/*ACKR1* axis may contribute to the tumorigenic effect of *BRCA1* MT TNBC.

### Sprouting angiogenesis through iCAFs-induced VEGF signaling might lead to resistance to combination therapy of cisplatin and bevacizumab in *BRCA1* MT TNBC patient

**Fig. 6:**
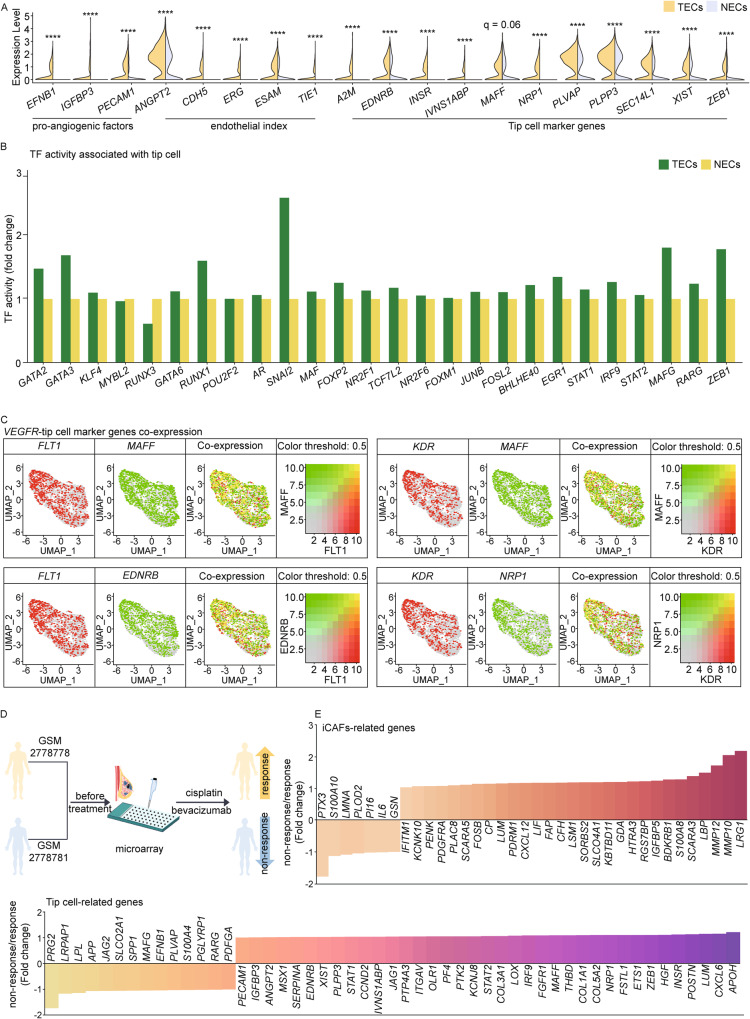
iCAFs-induced VEGF signaling elicits angiogenesis, leading to resistance to combination therapy of Cisplatin and Bevacizumab in TNBC *BRCA1* MT. **A** Violin plots illustrate that the expression levels of pro-angiogenic factors, endothelial indices, and tip cell marker genes between TECs and NECs. The expression level in TECs was mostly higher than in NECs. **B** Bar graphs display that the TF activity of tip cell markers in TECs was higher than in NECs. **C** Combined feature plots indicate high co-expression of *VEGFR* and tip cell marker genes, including *MAFF* or *EDNRB,* in the TECs cluster. **D** Schematic diagram shows two TNBC *BRCA1* MT expression data prior to neoadjuvant therapy combined with cisplatin and bevacizumab (top). **E** Bar graphs represent the fold change of microarray value associated with iCAFs and tip cell genes (top and bottom, respectively). In non-response patients, iCAFs and tip cell genes were higher than in response patients. *q*-values in **A** were obtained using the ‘p.adjust’ function in R (****q < 0.0001).

**Fig. 7:**
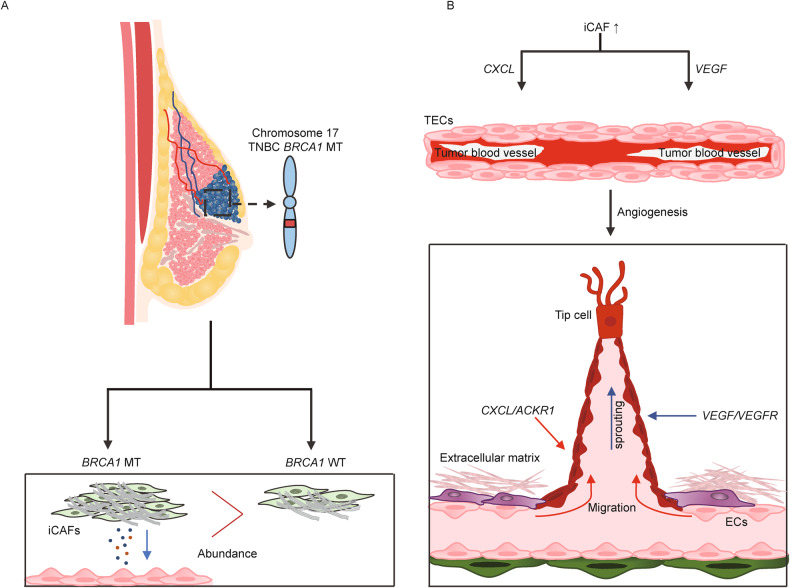
Schematic diagram of sprouting angiogenesis through iCAFs in TNBC *BRCA1* MT. **A** TNBC *BRCA1* MT germline mutation enriched the iCAF phenotype compared to WT. **B** iCAFs mainly secrete *CXCL* and *VEGF* to TECs, which interact with *ACKR1*, *FLT1*, and *KDR,* respectively. *CXCL*/*ACKR1* axis drives vascular stalk formation and TECs migration by inducing diverse angiogenic effects, including blood vessel loosening ECM remodeling. Additionally, *VEGFA*/*VEGFR* axis triggers the differentiation of TECs into tip cells, leading to sprouting angiogenesis.

Based on the outgoing signal of *VEGFA* from fibroblasts to TECs and the causal relationship between *VEGF* and tumor angiogenesis [39, 40], we investigated the expression levels of diverse genes associated with pro-angiogenic factors and the endothelial index between TECs and NECs in *BRCA1* MT TNBC patients. TECs exhibited higher expression levels of pro-angiogenic factor genes *EFNB1* and *PECAM1* and the endothelial index genes *CDH5* and *ESAM*. Interestingly, we observed that the expression levels of genes related to tip cells, an important component of sprouting angiogenesis, were upregulated in TECs compared to NECs (Fig. 6A). TF activity associated with tip cells, including *STAT1*, *MAFG*, and *ZEB1*, was also higher in TECs (Fig. 6B). Next, we examined whether these TECs exhibit co-expression of *VEGFR* and tip cell genes simultaneously using uniform manifold approximation and projection (UMAP) plots. The yellow color of the merged plots showed that *FLT1* and *KDR* had high co-expression with tip cell TF or genes, including *ZEB1*, *MAFF*, *EDNRB*, and *NRP1*, suggesting that TECs may promote sprouting angiogenesis via incoming VEGFA signaling (Fig. 6C). Finally, we found that *BRCA1* MT TNBC patient with relatively high expression levels of iCAFs and tip cell genes showed no response to neoadjuvant chemotherapy, including cisplatin and bevacizumab (Fig. 6D, E). Taken together, our data show that sprouting angiogenesis is activated through VEGF signaling between iCAFs and TECs, which might contribute to insensitivity to anti-angiogenic chemotherapy in *BRCA1* MT TNBC (Fig. 7A, B).

## Discussion

Although TNBC accounts for 15–20% of breast cancer cases, which have the highest incidence rate in women [5, 41], attempts to increase the survival rate of patients continuously have been performed due to the highest malignancy and lack of well-defined molecular targets. The *BRCA1* mutation appears frequently in TNBC patients, and its origin is somatic or germline mutation [42]. While many studies have explored the differences between somatic and germline mutation of *BRCA1/BRCA2* in TNBC [42–44], there is a lack of research focusing on the comparison between only *BRCA1* somatic and germline mutation. The proportion of *BRCA1* germline mutation patients was also higher than somatic mutation in TNBC [45]. So, we performed a single-cell analysis focusing on *BRCA1* germline mutation patients compared with cases without *BRCA1* mutation. While we confirmed that iCAFs were more abundant than WT in the context of *BRCA1* germline mutation, an analysis needs to be studied to see whether a consistent tendency is observed in somatic mutation.

A recent study on the molecular mechanism of *BRCA* mutations and CAF in pancreatic cancer reported enhancement of clusterin in *BRCA1* or *BRCA2* mutant CAF in a heat shock factor 1-dependent manner [46]. The research also observed that when pancreatic stellate cells were treated with conditioned media from *BRCA2*-deficient fibroblast cells, the RNA level of *Pdl1* associated with T cell immune suppression was increased. *Pdl1* was also found to be regulated in a heat shock factor 1-dependent manner. In contrast, our single-cell analysis showed nearly absent heat shock factor 1 expression in both TNBC *BRCA1* MT and WT, and clusterin expression was notably higher in the *BRCA1* WT fibroblasts than in MT (Lee et al., unpublished data). These differences may be a result of the tumor microenvironment heterogeneity of diverse cancer types and mutation types such as *BRCA1* or *BRCA2*. Furthermore, a study that tracked the evolution of CAFs after injecting TNBC 4T1 fibroblasts into BALB/c mice noted a decline in the proportion of *Pdpn*-positive CAFs and a rise in S100A4-positive CAFs in a time-dependent manner [47]. In addition, TNBC patients with *BRCA* mutations and a low S100A4/PDPN ratio exhibited shorter recurrence-free survival compared to those with *BRCA* WT. Nevertheless, the precise molecular mechanisms governing the S100A4/PDPN/BRCA mutation axis remain unresolved. Therefore, studies of *BRCA* mutation and CAFs, including TNBC and other types of cancer, are still indispensable.

Investigations about the role of iCAFs in TME of TNBC patients with *BRCA1* mutation have not yet been conducted. We identified the heightened presence of iCAFs in the TNBC *BRCA1* MT. Previous studies reported elevated inflammatory response in TNBC patients with *BRCA1* mutation [48]. So, we hypothesized that the elevated inflammatory response might contribute to the increased proportion of iCAFs in TNBC with *BRCA1* mutation. Nonetheless, further investigation is needed to identify which factor or pathway directly enhances iCAFs phenotype in patients with *BRCA1* mutation. In addition, CAFs are highly plastic cells with diverse origins, such as normal fibroblasts, ECs, or macrophages [49]. However, our bioinformatics analysis had limitations in identifying the precise origin of iCAFs in *BRCA1* mutation patients.

scRNA-seq analysis showed the pivotal role of iCAFs in modulating ECs through outgoing signaling of CXCL and VEGF under patients with TNBC *BRCA1* mutation. In particular, cell–cell communication analysis revealed that iCAFs mainly send CXCL and VEGF signaling to TECs. TECs play an important role in tumor growth by building up the inner layer of blood vessels. Based on the characteristics of vascular leakage, increased interstitial fluid pressure, and low rate of blood flow, TECs may be involved in tumor angiogenesis and metastasis in diverse types of cancer [50–52]. Our data also suggest the sprouting angiogenic role of TECs via inducing vascular stalk formation and migration, which is likely due to CXCL and VEGF signaling in TNBC with *BRCA1* mutation.

The CXCL subfamily is prominently associated with inflammation and immune response control through the migration of leukocytes [53]. Moreover, they regulate tumor cell proliferation and angiogenesis, thereby accelerating cancer development. The CXCL family is highly upregulated in cancer and strongly associated with metastasis and chemoresistance. Angiogenesis and metastasis are fundamental characteristics of cancer, and *CXCL* plays a crucial role in cancer research, particularly in TNBC [54]. The CXCL subfamily triggers the production of pro-angiogenic factors or attaches to chemokine receptors on endothelial cell surfaces, promoting angiogenesis [55]. Also, CXCL signaling promotes leukocyte infiltration in various cancer types through its interaction with *ACKR1* in TECs [56]. This interaction triggers *MMP* secretion, which contributes to EC migration and metastasis. Our findings align with this pattern, as the CXCL family in iCAFs strongly interacts with *ACKR1* in TECs, leading to the enhancement of angiogenesis-related genes such as ECM remodeling and immune adhesion. Thus, targeting *CXCL/ACKR1* axis of iCAFs is crucial for angiogenesis inhibition in TNBC with *BRCA1* mutation.

*VEGF*, a major angiogenic factor in breast cancer, promotes angiogenesis and increases vascular permeability [57]. Elevated *VEGF* expression has also been linked to diminished responses to chemotherapy or tamoxifen in patients with advanced breast cancer [58]. *VEGF* binds to *VEGFR* in ECs and triggers angiogenesis. When angiogenesis occurs in response to *VEGF*, tip cells play a vital role in angiogenesis in response to *VEGF*. Tip cells interpret environmental cues and guide the growth of new sprouts. Moreover, tip cells establish connections with different sprouts to form a functional vascular network. Interestingly, our analysis revealed high *VEGFR* expression in TECs, along with elevated activity of tip cell markers and TF. Considering these findings, our data suggest that *VEGF* released from iCAFs might induce sprouting angiogenesis by building up the inner layer of blood vessels, leading to tip cell extension.

Our analysis of clinical data comparing two TNBC *BRCA1* MT patients revealed that patients who did not respond to neoadjuvant chemotherapy exhibited a high proportion of iCAFs and angiogenesis-related genes compared to patients with sensitivity to chemotherapy. Patients with elevated levels of angiogenic genes, such as tip cell marker genes, exhibit poor responses to anti-angiogenic therapy or unfavorable outcomes. Additionally, many studies have indicated that patients with increased iCAFs-related genes do not respond to antiangiogenic drugs in diverse types of cancers, including pancreatic ductal adenocarcinoma and TNBC [59, 60]. Indeed, imatinib, a well-known anticancer drug for chronic myeloid leukemia, has also been shown to inhibit the iCAFs marker *PDGFRA* to suppress angiogenesis in cervical carcinoma [61]. These indicate that the iCAFs signature could be a useful biomarker in TNBC *BRCA1* MT patients for enhancing the efficacy of anti-angiogenic therapy [62, 63]. In conclusion, our data suggest that TNBC patients with *BRCA1* MT are characterized by an enhanced iCAFs phenotype, and targeting iCAFs signaling in those with a relatively higher signature could overcome the insensitivity to anti-angiogenic chemotherapy.

## Materials and methods

### Spatial transcriptome

To analyze the spatial transcriptome, relevant data were obtained from the public dataset GSE210616. This dataset comprises information from 22 patients diagnosed with TNBC. The analysis was conducted using the ‘Seurat’ package (version 4. 3. 1) in R. Tissue image PNG files were imported into R using the ‘Read10X_Image’ function. Subsequently, preprocessed spatial transcriptomics data matrices in H5 format were loaded through the ‘Load10X_Spatial’ function. Data normalization was carried out using the ‘NormalizeData’ function, and the top 2000 highly variable genes were identified utilizing the ‘FindVariableFeature’ function. The PCA was performed for cluster separation facilitated by the ‘RunPCA’ function. Subsequent graph clustering based on nearest neighbors was performed using the ‘FindNeighbors’ function with dimensions 1:30. The cell subtypes were delineated using the ‘FindClusters’ function, with a resolution of 0.5. Finally, the expression matrix was confirmed through ‘RunUMAP’ using dimensions 1:30.

The scoring of signatures for CAF genes and NCAF genes was calculated utilizing the ‘Addmodulescore’ function. The list of CAF genes and NCAF genes is outlined in more detail in Supplementary Table 1. The spatial feature expression plots were visualized using the ‘SpatialFeaturePlot’ function. The highest expression level of the fibroblast gene *SPARC* was determined using the ‘Vlnplot’ function, which was designated as the fibroblast-dominant cluster. A scatter plot was generated using the ‘FeatureScatter’ function to illustrate the relationship between the CAF or NCAF signature scores and the dominant clusters based on the fibroblast-dominant cluster. The three groups were divided by the *r*-value difference between CAF and NCAF, and the standards are as follows. (CAF high group: *r*-value difference > 0.1, NCAF high group: r-value difference < −0.1, No difference group: −0.1 < *r*-value difference < 0.1).

### Comparison of CAF gene expression between TNBC and non-TNBC

The ‘Breast Cancer Integrative Platform (BCIP)’ (http://www.omicsnet.org/bcancer/) website was used comparing gene expression between TNBC and non-TNBC groups. Cancer vs cancer analysis was conducted, and TNBC vs non-TNBC sample subgroups were created based on ‘Metabric’ dataset. The expression values of individual CAF genes were then visualized using box plots, along with corresponding *p*-values denoting significance.

### Kaplan–Meier survival analysis

For CAF-related genes and TNBC-specific DMFS and OS analysis, Kaplan–Meier survival curves were generated using breast cancer microarray data from 275 and 216 patients in the KM Plotter database (https://kmplot.com/analysis/). Two analyses of the CAF signature were conducted using CAF marker genes. The list of these marker genes is provided in more detail in Supplementary Table 2. For both analyses of CAF signature, the ‘Use Multiple Genes’ function in KM Plotter was used.

### Acquisition of scRNA-seq data

The findings of the single-cell analysis were obtained from the public dataset GEO database. scRNA-seq data of TNBC *BRCA1* mutation patients (*n* = 4) and TNBC *BRCA1* wild-type patients (*n* = 4) were obtained from the GSE161529 dataset. The scRNA-seq data were processed using the R package ‘Seurat’. Initially, expression matrices were loaded using the ‘Read10X’ function. Quality control measures were applied to remove poor-quality cells based on the criteria: number of genes detected per cell (nFeature), total molecule count per cell (nCount), and percentage of mitochondrial genes (percent_MT). Subsequently, doublets were identified and removed using ‘doubletFinder_v3’ (version 2. 0. 3). Data normalization was carried out using ‘LogNormalize’, and the top 2000 most highly variable genes were identified using ‘FindVariableFeatures’. Integration of the data was achieved through ‘IntegrateData’.

### Processing of the scRNA-seq data

PCA was performed to determine the principal components (PCs). Based on these results, ‘RunPCA’ was executed to estimate UMAP using the top 30 significant PCs. A shared nearest-neighbor graph was constructed using ‘FindNeighbors’ on the UMAP coordinates. Clusters were then identified by refining SNN modularity using ‘FindClusters’.

Specific information regarding the quality control and doublet percentages can be found in Supplementary Table 3. The Seurat packages facilitate the integration of unique Seurat objects into a single integrated object. The integration anchors are estimated using ‘SelectIntegrationFeatures’ and ‘FindIntegrationAnchors’. Datasets were combined using estimated anchors via ‘IntegrateData’. After merging, data were normalized using ‘NormalizeData’. Cluster marker genes showing a log fold change (logFC) exceeding 0.25 compared to other clusters were identified using ‘FindAllMarkers’. Cluster annotation was performed by comparing selected reference genes.

### DEG analysis

After subclustering fibroblasts in TNBC *BRCA1* MT and WT patients using ‘RenameIdents’, DEG analysis between iCAFs and myCAFs was conducted. DEGs were extracted using ‘FindMarkers’ with a logFC value exceeding 0.58. Expression levels of these DEGs were displayed using ‘Dimplot’ and ‘Featureplot’ with reference genes. A heatmap of fibroblast DEG expression was generated using ‘DoHeatmap’. A new Seurat object incorporated all fibroblast genes, and enrichment scores based on Hallmark pathways were visualized using ‘dittoPlot’. Average expression levels were shown using ‘VlnPlot’ based on calculated single-cell average expression levels with ‘AddmoduleScore’. Growth factor and cytokine expression comparison of MT and WT in fibroblasts or all clusters were achieved through ‘DotPlot’.

ECs were further divided into *ACKR1* high TECs, *ACKR1* low TECs, *ACKR1* high NECs, and *ACKR1* low NECs based on *ACKR1* expression levels. The division was verified by checking TEC marker genes and *ACKR1* expression levels using ‘DotPlot’. To distinguish between TECs, NECs, and ECs based on *ACKR1* expression, we employed the ‘FeaturePlot’ function to visualize UMAP. Subsequently, extract DEGs from the clusters based on *ACKR1* expression by using the ‘FindMarkers’. Finally, visualize the data with a volcano plot using ‘GraphPad Prism’ (version 9. 5. 1).

GO pathway enrichment analysis was conducted using the ‘escape’ function and visualized with ‘dittoDotPlot’. Co-expression plots were generated using the ‘FeaturePlot’ function. To visualize the coexpression of two features, we scaled and blended the expression values. Also, set the maximum cutoff of each feature’s value to 1. To calculate *q*-value, the function ‘p.adjust’ was used.

### Gene set enrichment analysis

The R package ‘escape’ (version 1. 10. 0) was used to perform single-cell gene set enrichment analysis. Single-cell gene set enrichment analysis scores were calculated for individual cells based on Hallmark gene sets (‘H’) and GO biological process in ontology gene set (‘C5’) from the MSigDB database, which was obtained using the ‘getGeneSets’ function. The ‘enrichIt’ function was used to input the scRNA-seq counts data and each pathway data. Visualization of each gene set was achieved using ‘dittoVlnPlot’ and ‘dittoDotPlot’ functions. To check for significance, the ‘stat_compare_means’ function of the ‘ggpubr’ package was used.

### Transcription factor activity analysis

This analysis utilized the curated collection of TFs known as ‘DoRotheEA’ (version 1. 12. 0). Human data were retrieved using the ‘get_dorothea’ function, and to use the Weighted Mean method, the ‘run_wmean’ function was used. This method ensures that the ‘wmean’ is first multiplied by each target feature with its associated weight and then summed to the average of the enrichment scores. Subsequently, the data were scaled, leading to the identification of the top transcription factors with variable means across the clusters.

### Cell–cell communication analysis

For cell–cell communication analysis, fibroblasts and seven other cell types in TNBC *BRCA1* MT were examined using the R package ‘CellChat’ (version 1. 6. 1). Outgoing signaling of CXCL, VEGF, CCL, FGF, and MIF was visualized using a chord diagram, highlighting the intercellular communication patterns. For chord diagram visualization, the ‘netVisual_chord_gene’ function was used. Subsequently, the ‘plotGeneExpression’ function from the ‘SeuratWrappers’ package was used to assess the distribution of CXCL and VEGF signaling gene expression. The gene expression distribution of signaling genes associated with L–R pairs or signaling pathways were visualized as violin plots.

### Bulk sequencing of TNBC patients with neoadjuvant therapy

We utilized data from the GSE103688 dataset, specifically GSM2778778 and GSM2778781 with *BRCA1* mutation. GSM2778778 was categorized as ‘response’, while GSM2778781 was defined as ‘non-response’ in neoadjuvant chemotherapy. A gene expression analysis was performed based on fold change values, and the comparative data was visualized using ‘GraphPad Prism’ (version 9. 5. 1).

### Supplementary information

Supplemental material

## Data Availability

The Visium data can be accessed in the Gene Expression Omnibus under accession number GSE210616. The dataset for single-cell RNA sequencing is available under GSE161529. Additionally, the bulk sequencing data utilized in this study were sourced from the GSE103688 dataset, specifically GSM2778778 and GSM2778781.
