# Supplementary material for: BRCA1 mutation promotes sprouting angiogenesis in inflammatory cancer-associated fibroblast of triple-negative breast cancer
Source: Cell Death Discov. 2024 Jan 5;10:5. doi: 10.1038/s41420-023-01768-5 (PMC10770063; doi:10.1038/s41420-023-01768-5)
Supplement: Supplementary file 1 — Supplemental material [file 41420_2023_1768_MOESM1_ESM.docx]

**
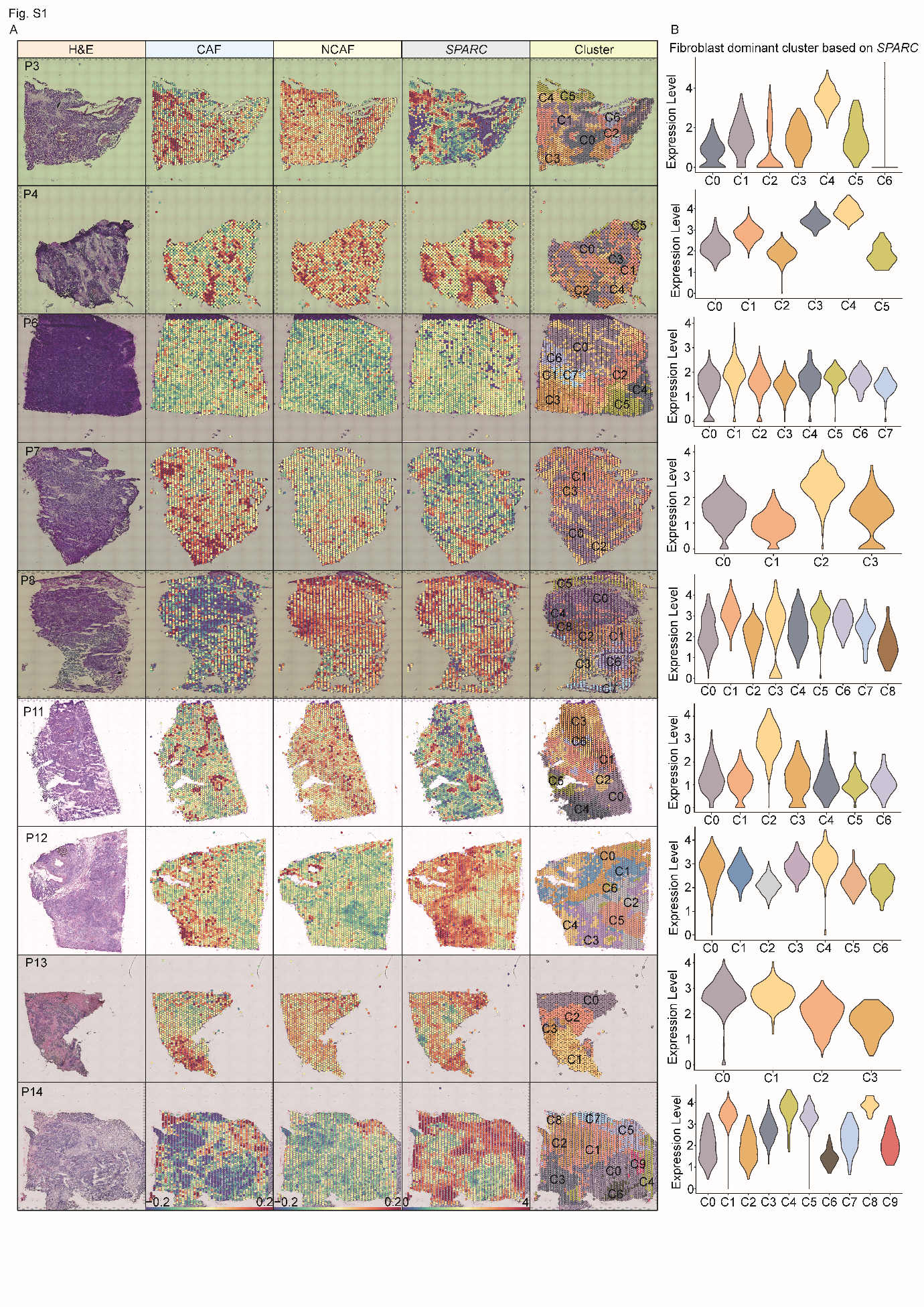
**

**
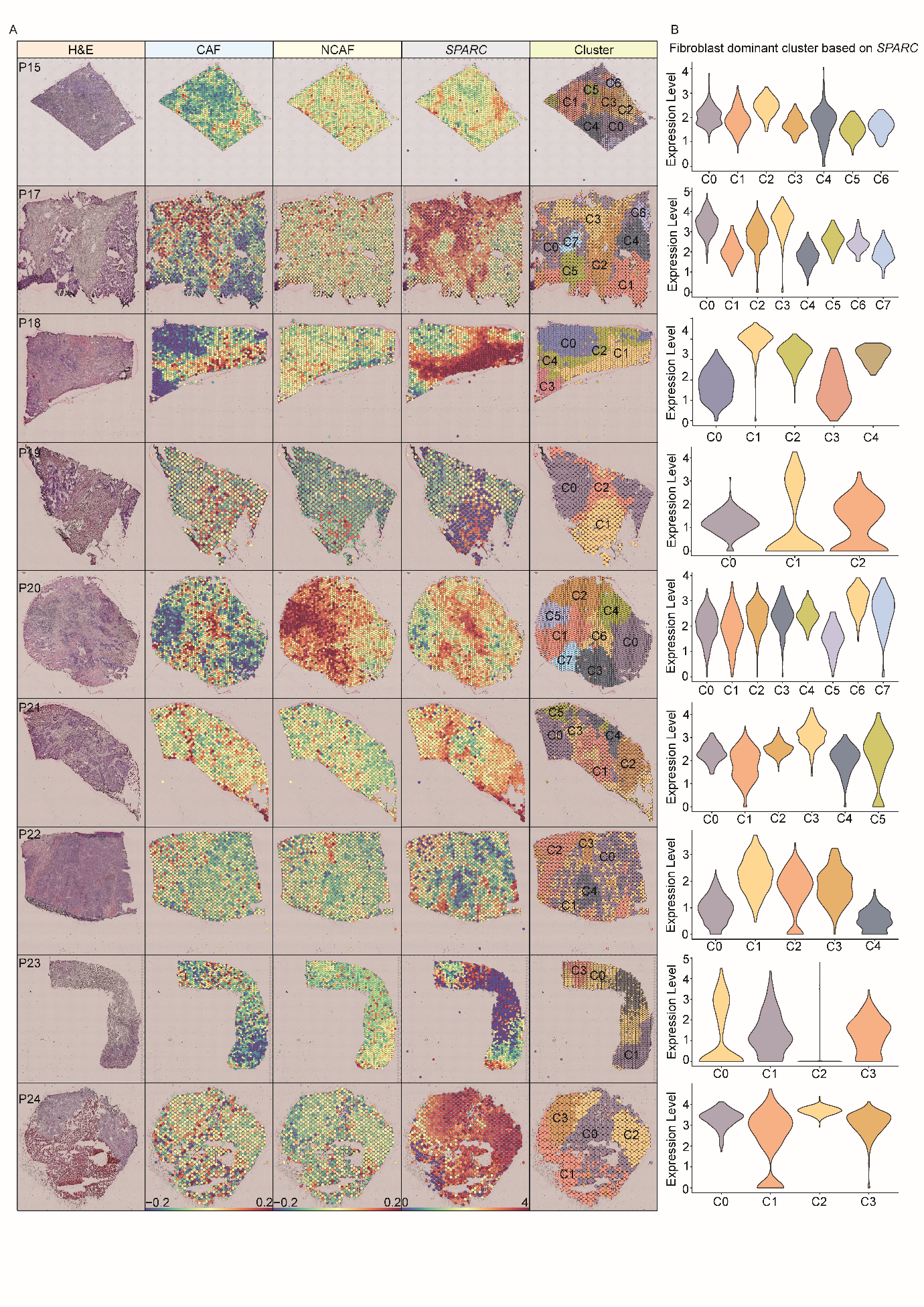
**

**
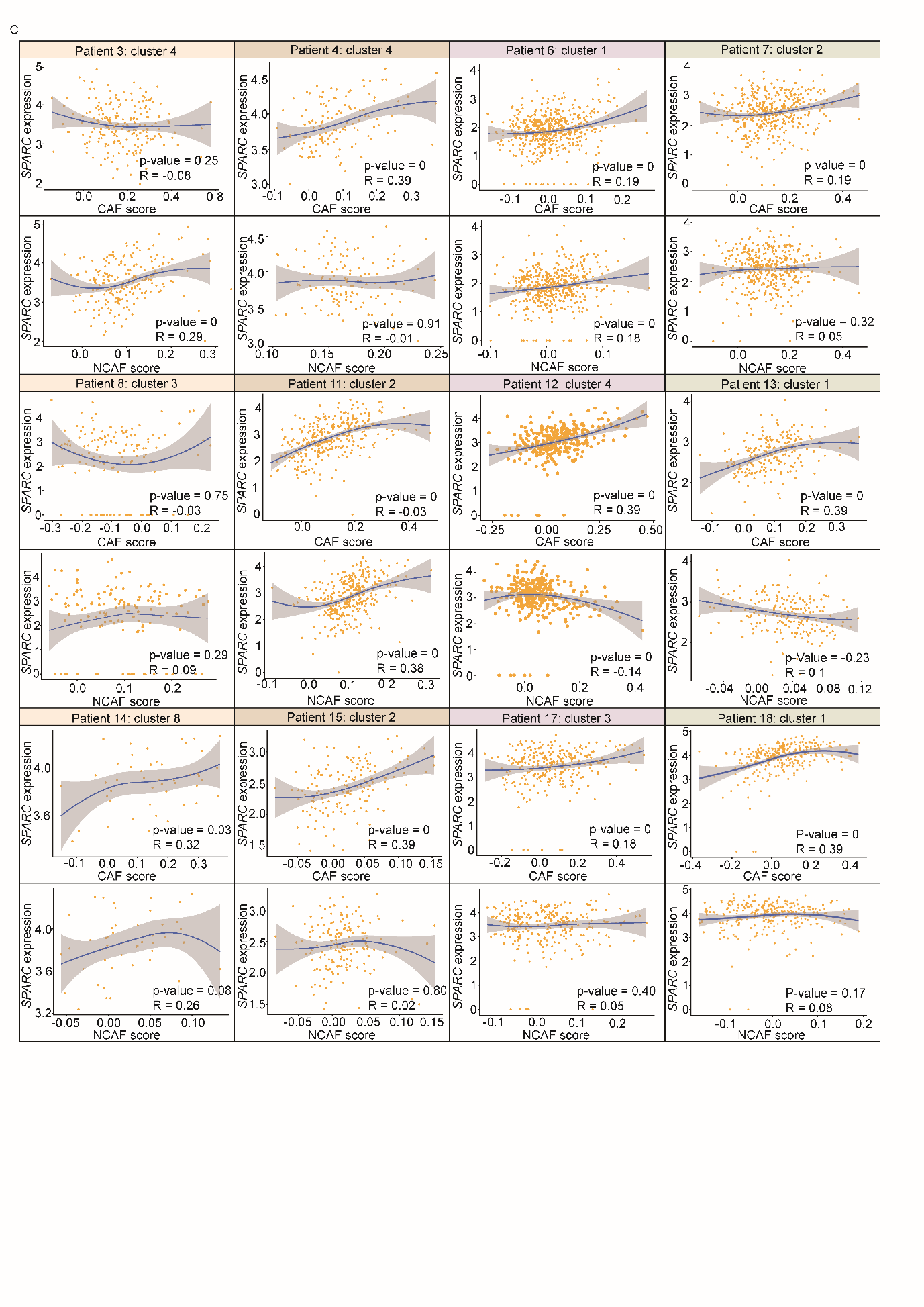
**

**
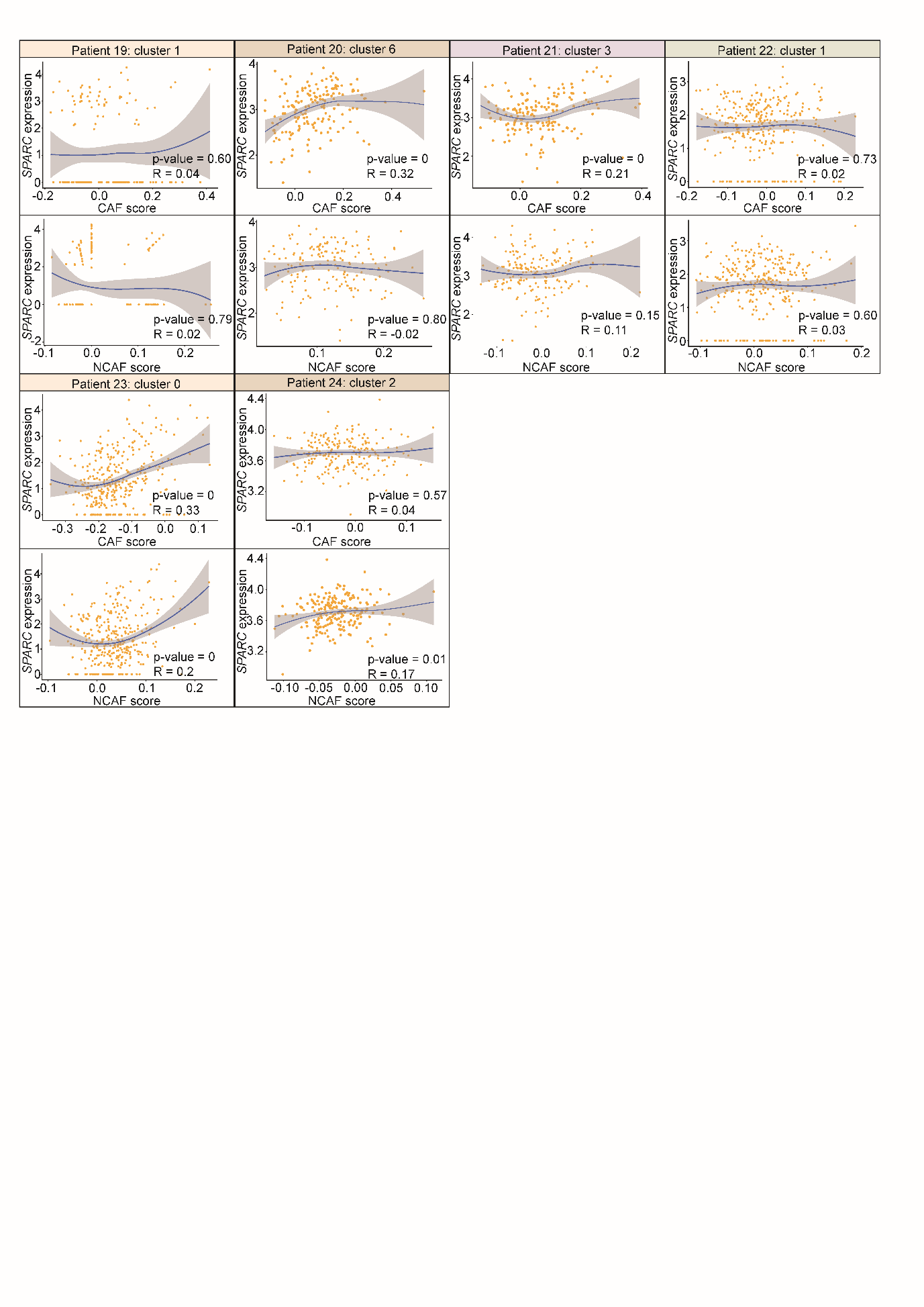
**

**Supplementary Fig. 1 TNBC patients show abundance of CAF rather than NCAF signature A** Visium spatial gene expression data illustrates that the expression patterns of CAF and NCAF signature based on fibroblast marker gene ‘*SPARC’.* Patients who had higher CAF score compared to NCAF were named P4, P7, P12, P13, P15, P17, P18, P20, P21, and P23 (CAF high group). On the other hand, NCAF score was significantly higher at P3, P8, P11, and P24 (NCAF high group). The P6, P14, P19 and P22 showed no difference in signature between CAF and NCAF (No difference group). **B** Violin plots display fibroblast-dominant clusters categorized by *SPARC* expression for each patient. The clusters with the highest prominence in each patient are accentuated in yellow. **C** Scatter plots depict the relationship between CAF or NCAF signatures and *SPARC* within the identified fibroblast-dominant cluster from


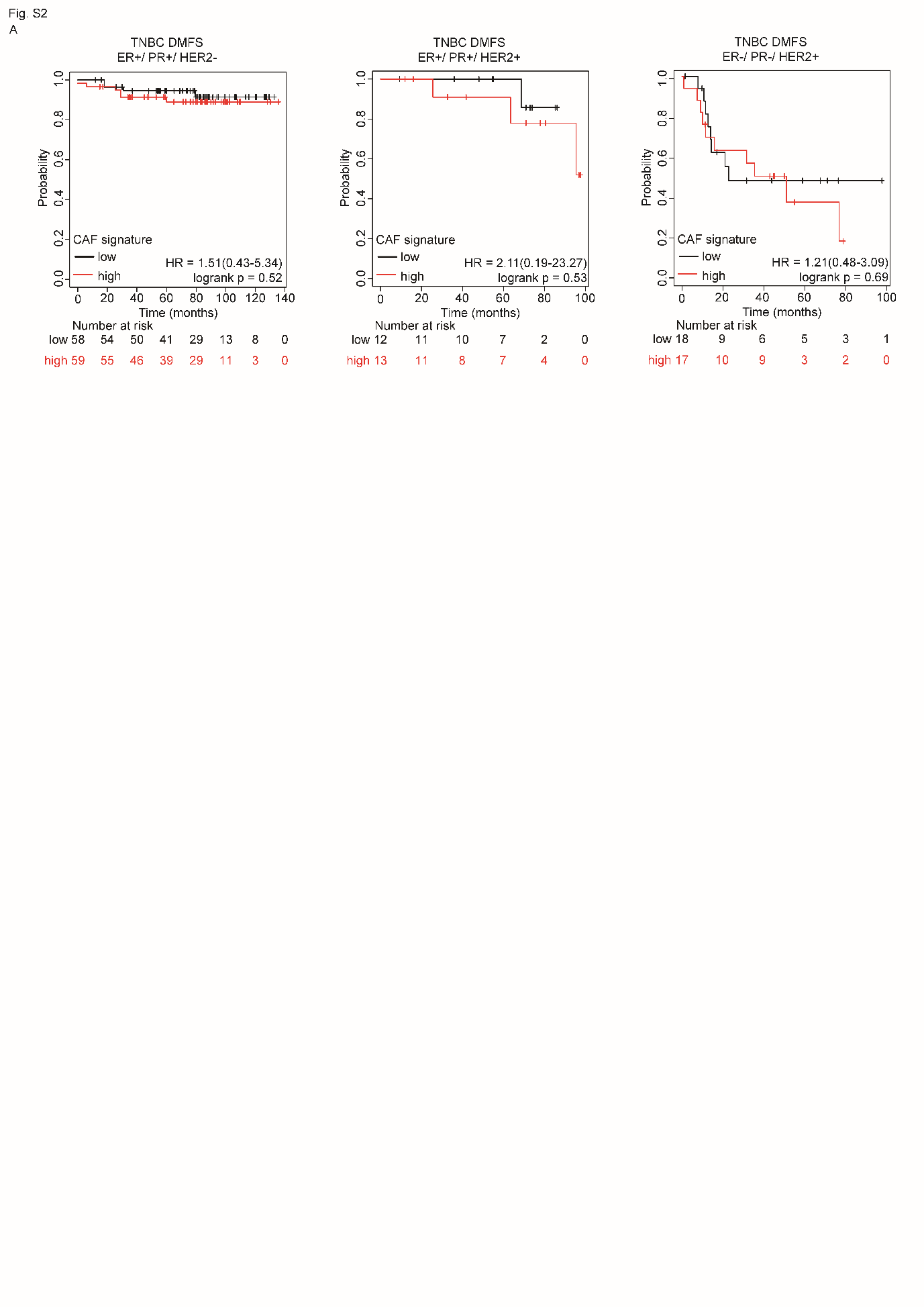


**Supplementary Fig. 2 CAF signature is not associated with inferior outcome in DMFS of diverse breast cancer subtypes** Kaplan–Meier curves display non-TNBC breast patient subtypes do not exhibit significant differences in the distant metastasis-free survival (DMFS) between patients with CAF signature high and low groups. Graphs represent ER+/PR+/HER2- (left), ER+/PR+/HER2+ (middle) and ER-/PR-/HER2+ (right) subtypes respectively.


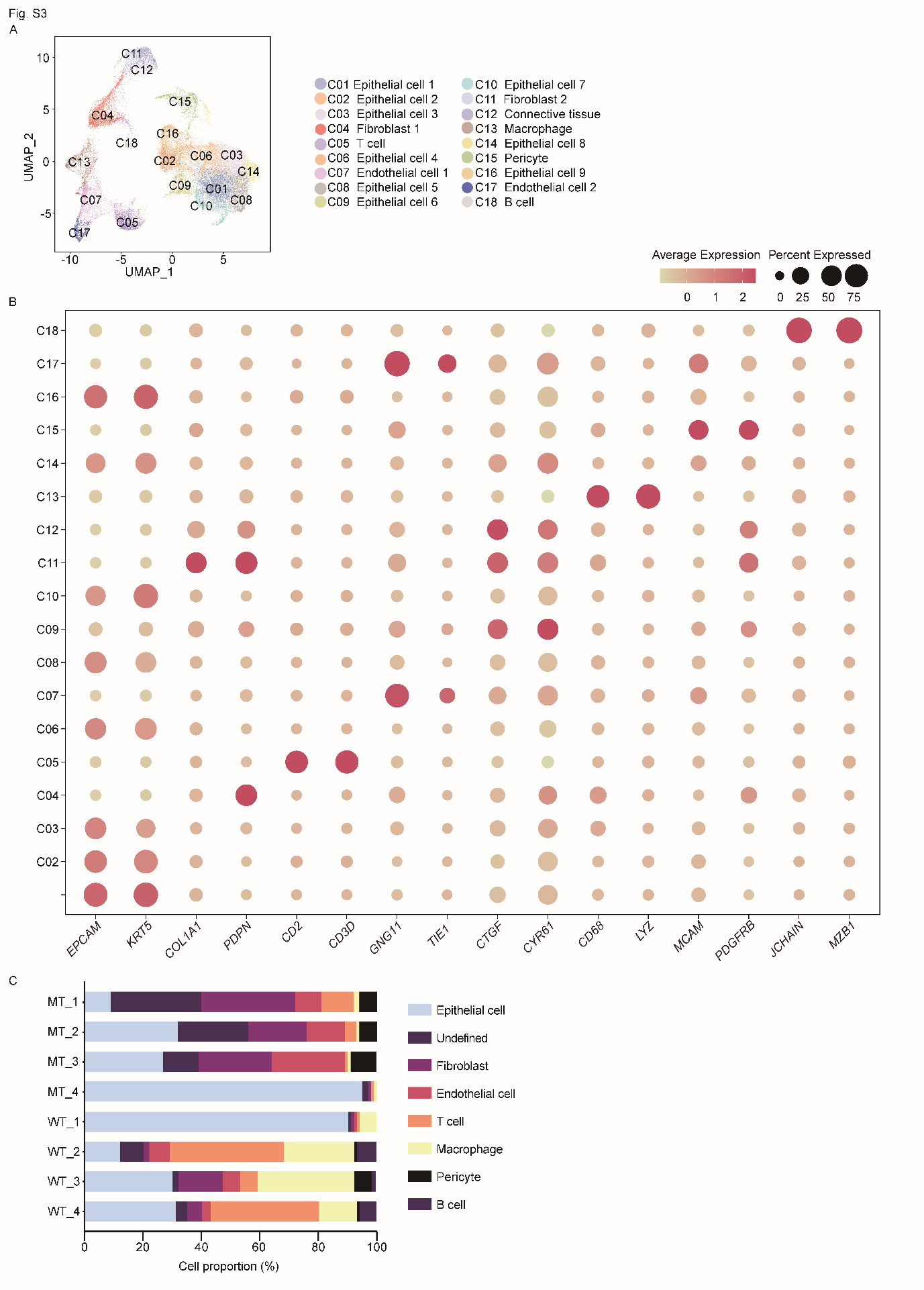


**Supplementary Fig. 3 Characteristics of cellular state in TNBC *BRCA1* MT and WT. A** An integrated UMAP plot illustrates total 18 distinct clusters. **B** Dot plot shows dot size representing the marker expressed percentage of cells and the color scale means the average marker expression in TNBC patients with *BRCA1* MT and WT total clusters in **A**. **C** Bar graph shows the proportion of major cell types and the heterogeneity of cell distribution in in total 8 TNBC patients with *BRCA1* MT and WT.

**Supplementary Table 1. Gene list of CAF and NCAF used in spatial transcriptome**

| **CAF genes** | *ANKRD1* | *B3GALT2* | *CHI3L1* | *COL11A1* | *CTHRC1* | *EVI2B* |
| --- | --- | --- | --- | --- | --- | --- |
|  | *ICAM1* | *ITGA11* | *LPXN* | *MCTP2* | *MFAP5* | *MME* |
|  | *OXTR* | *PTPRB* | *ST6GALNAC5* | *SULF1* | *SYT14* | *TBC1D2* |
|  | *THBS2* | *TLR4* | *TNFSF4* |  |  |  |
| **NCAFgenes** | *ACPP* | *ADAMTS8* | *ADGRG6* | *ANO4* | *A2M* | *BMP4* |
|  | *BRINP3* | *CCDC102B* | *CLU* | *COL14A1* | *FGF10* | *FLRT3* |
|  | *GAL* | *IGSF10* | *KLHL13* | *LAMP5* | *NOVA1* | *PDE3B* |
|  | *RASL11A* | *SLC51B* | *SLITRK6* | *TBX4* | *TMOD1* | *TNFRSF19* |

**Supplementary Table 2. Gene list of CAF used in KM Plotter**

| **CAF genes** | *ANKRD1* | *B3GALT2* | *CHI3L1* | *COL11A1* | *CTHRC1* | *EVI2B* |
| --- | --- | --- | --- | --- | --- | --- |
|  | *ICAM1* | *ITGA11* | *LPXN* | *MCTP2* | *MFAP5* | *MME* |
|  | *NOX4* | *OXTR* | *PTPRB* | *ST6GALNAC5* | *SULF1* | *TBC1D2* |
|  | *THBS2* | *TLR4* | *TNFSF4* |  |  |  |

**Supplementary Table 3. Quality control and doublet percentages information**

|  | MT_1 | MT_2 | MT_3 | MT_4 | WT_1 | WT_2 | WT_3 | WT_4 |
| --- | --- | --- | --- | --- | --- | --- | --- | --- |
| min.cells | 3 | 3 | 3 | 3 | 3 | 3 | 3 | 3 |
| min.features | 200 | 200 | 200 | 200 | 200 | 200 | 200 | 200 |
| percent.mt | <5 | <5 | <5 | <5 | <5 | <5 | <5 | <5 |
| PCA_npcs | 20 | 20 | 20 | 20 | 20 | 20 | 20 | 20 |
| UMAP_dims | 1:10 | 1:10 | 1:10 | 1:10 | 1:10 | 1:10 | 1:10 | 1:10 |
| doublet_pN | 0.25 | 0.25 | 0.25 | 0.25 | 0.25 | 0.25 | 0.25 | 0.25 |
| doublet_pK | 0.09 | 0.09 | 0.09 | 0.09 | 0.09 | 0.09 | 0.09 | 0.09 |
| doublet_PCs | 1:10 | 1:10 | 1:10 | 1:10 | 1:10 | 1:10 | 1:10 | 1:10 |
| nFeature_RNA | <6000 | <3500 | <2500 | <3000 | <3000 | <3000 | <2500 | <3000 |
| ncount_RNA | <40000 | <15000 | <10000 | <15000 | <15000 | <10000 | <7500 | <10000 |
| Variablefeatures  _nfeatures | 2000 | 2000 | 2000 | 2000 | 2000 | 2000 | 2000 | 2000 |
